# Supplementary material for: The Multi-Step Chain Extension for Waterborne Polyurethane Binder of Para-Aramid Fabrics
Source: Molecules. 2022 Nov 5;27(21):7588. doi: 10.3390/molecules27217588 (PMC9656495; doi:10.3390/molecules27217588)
Supplement: Supplementary file 1 [file molecules-27-07588-s001.zip › molecules-1968807-supplementary.pdf]

Supporting information

The multi-step chain extension for waterborne polyurethane binder of *para*-aramid fabrics

Table S1 Relative contributions of free, ordered and disordered H-bonded C=O in WPU.

Sample	Peak 1	Peak 2	Peak 3
	ordered	disordered	free
WPU-G	27	28	45
$3D_1$-G	31	44	25
$3D_2$-G	29	46	25
$3D_3$-G	39	26	35
$3D_4$-G	32	41	27
$3D_5$-G	31	46	23
$3T_2$-G	70	25	5
$3P_2$-G	65	20	15
$1D_2$-G	60	20	20
$2D_2$-G	63	21	16
$3D_2$-G_8L_2	62	22	16
$3D_2$-G_6L_4	48	32	20
$3D_2$-G_4L_6	57	20	23
$3D_2$-G_2L_8	58	27	15
$3D_2$-L	28	32	40

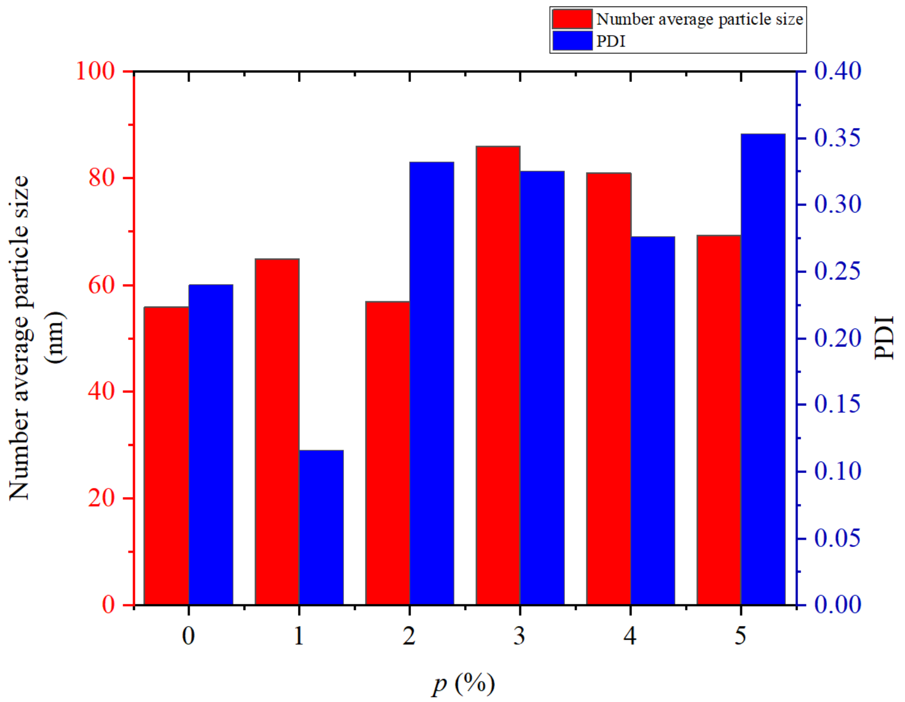

Figure S1. Effect of DMU content on the number-average particle size and PDI of WPU-G and 3D$_p$-G.

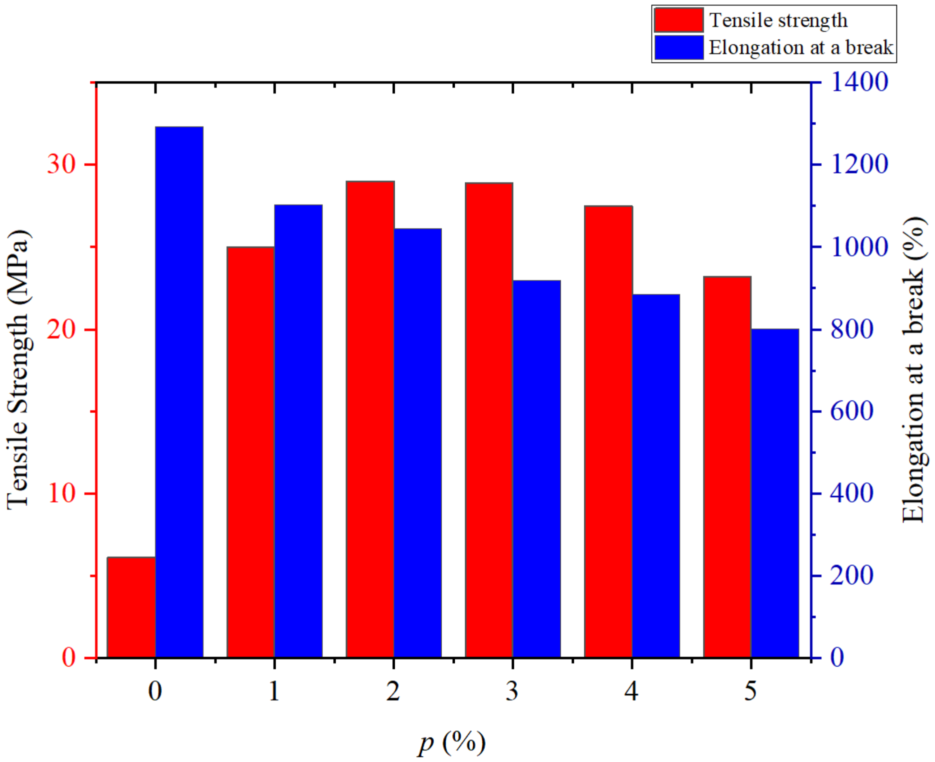

Figure S2. Effect of DMU content on tensile strength and elongation at break of WPU-G and 3D$_p$-G.

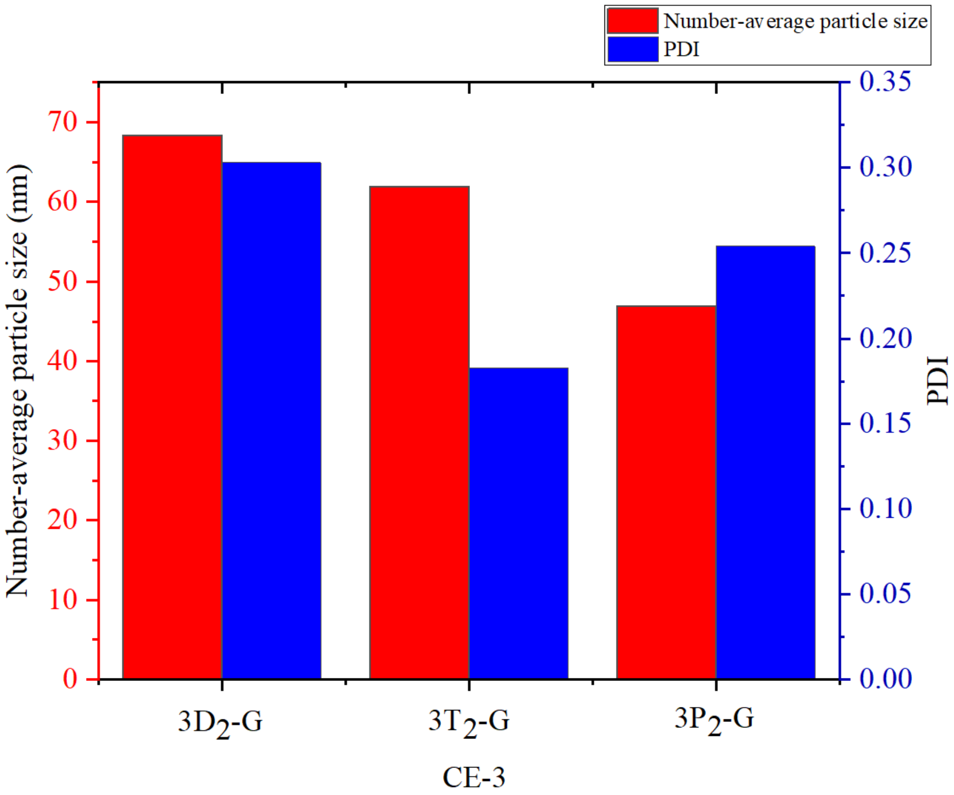

Figure S3. Number average particle size and PDI of $3D_2$-G, $3T_2$-G and $3P_2$-G.

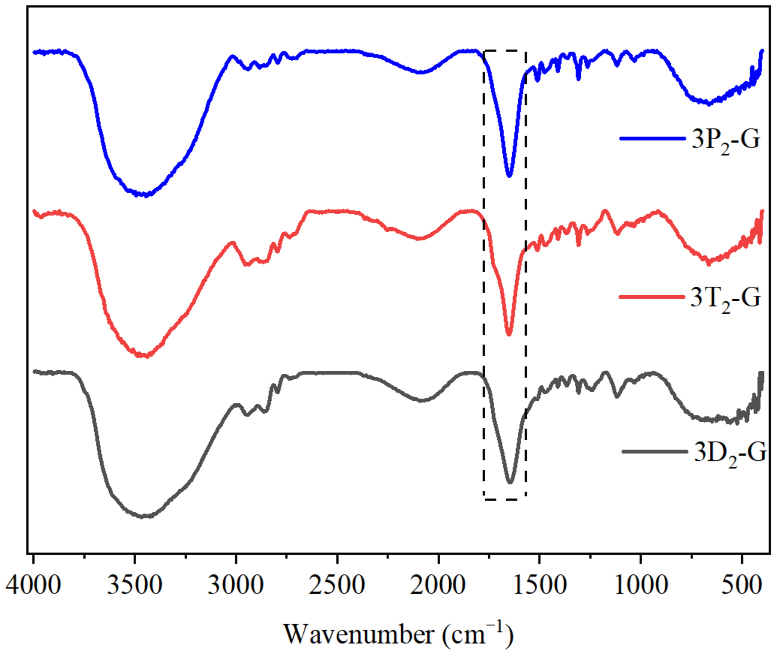

Figure S4. The FTIR spectra of $3D_2$-G, $3T_2$-G and $3P_2$-G.

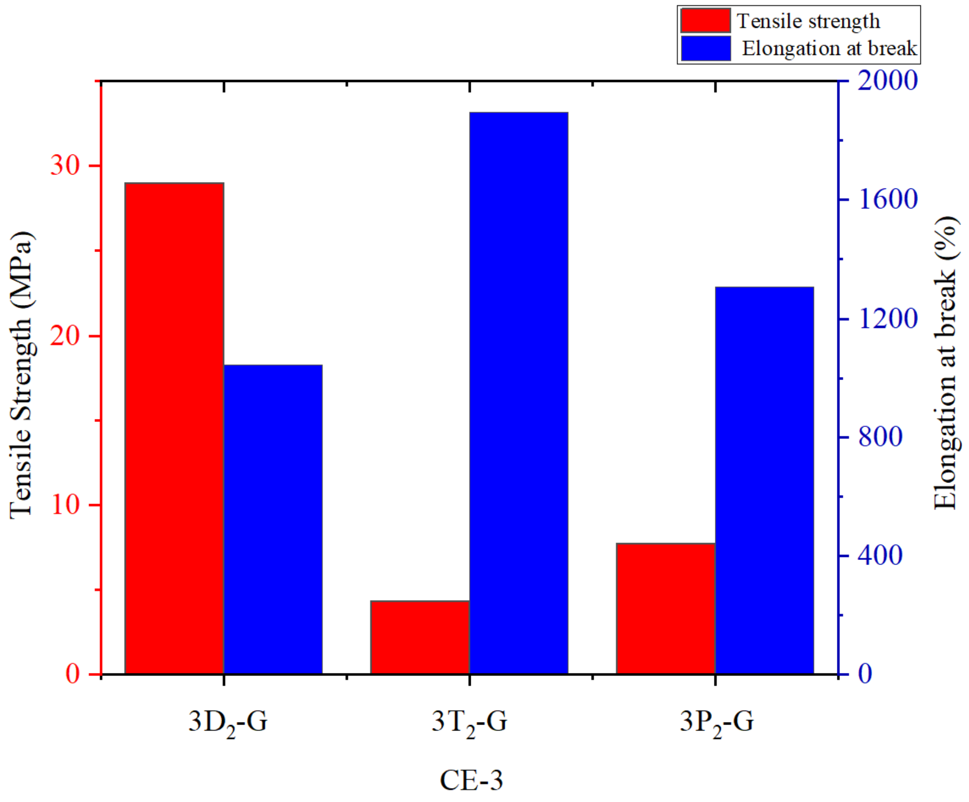

Figure S5. Tensile strength and the elongation at break of $3D_2$-G, $3T_2$-G and $3P_2$-G.

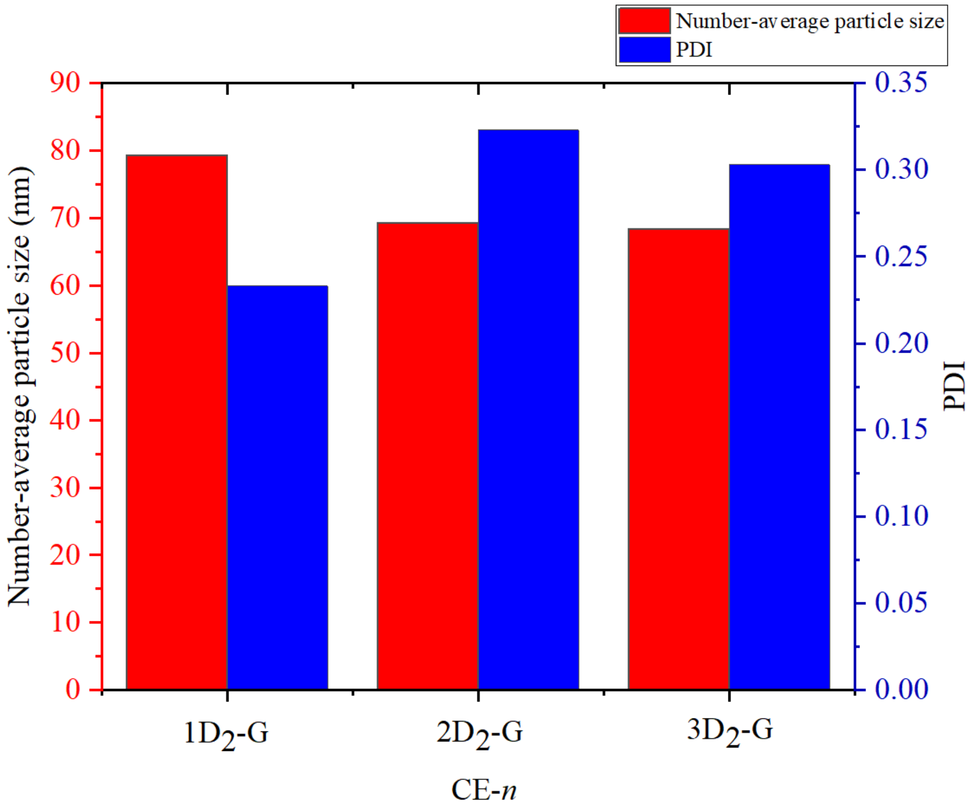

Figure S6. Number average particle size and PDI of $1D_2$-G, $2D_2$-G and $3D_2$-G.

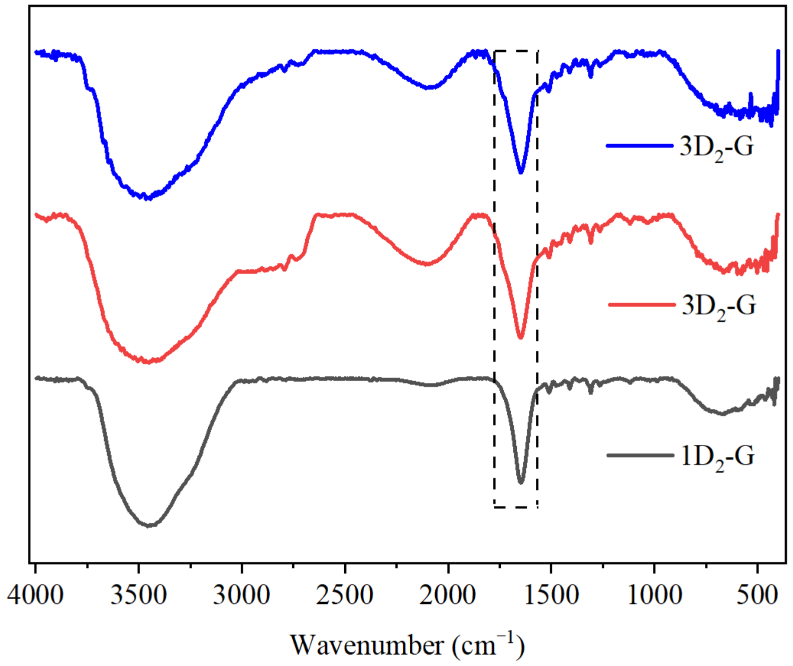

Figure S7. The FTIR spectra of 1D2-G, 2D2-G and 3D2-G.

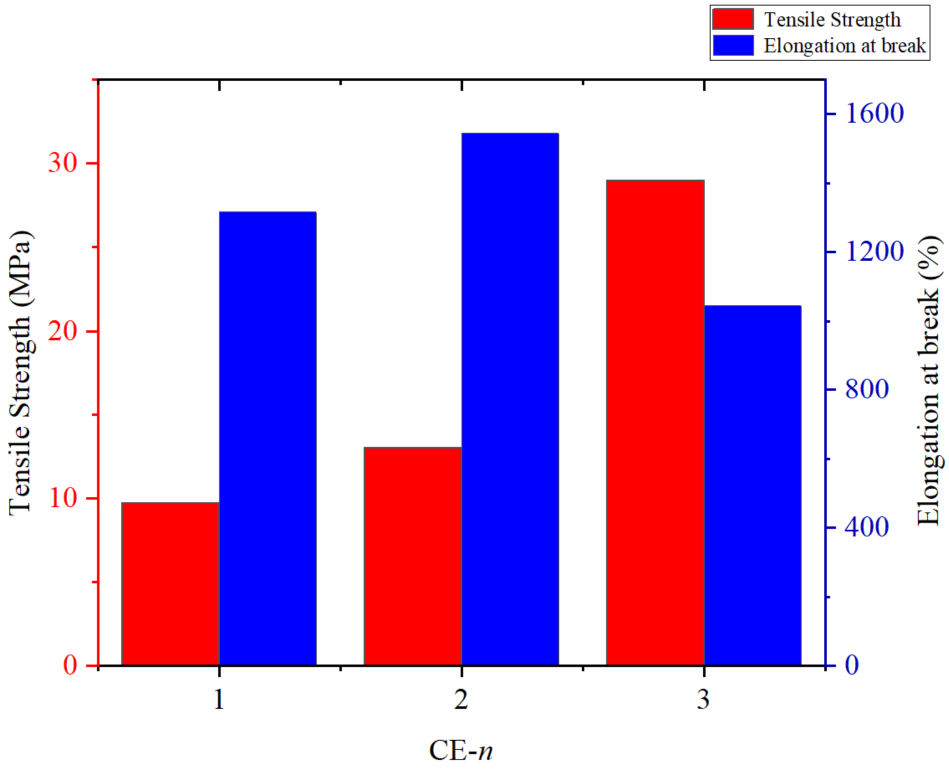

Figure S8. Tensile strength and the elongation at break of 1D2-G, 2D2-G and 3D2-G.

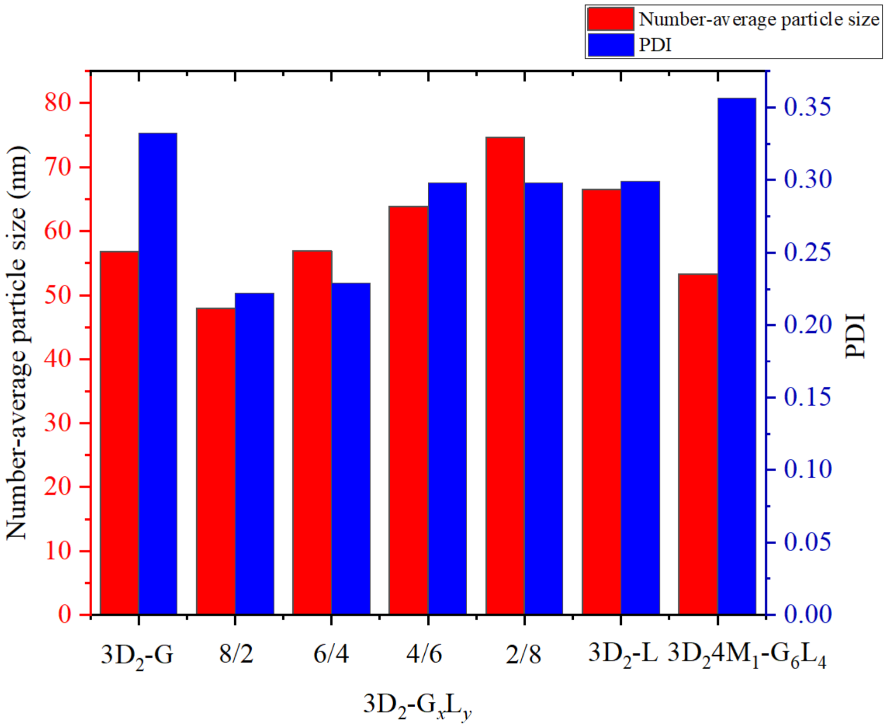

Figure S9. Effect of PTMG/PCL molar ratio (*x*/*y*) on number-average particle size and PDI of 3D2-G*x*L*y* samples.(where *x*/*y* were 8/2, 6/4, 4/6, and 2/8, respectively. For comparison purposes , also summarizes the results for 3D2-G and 3D2-L.)

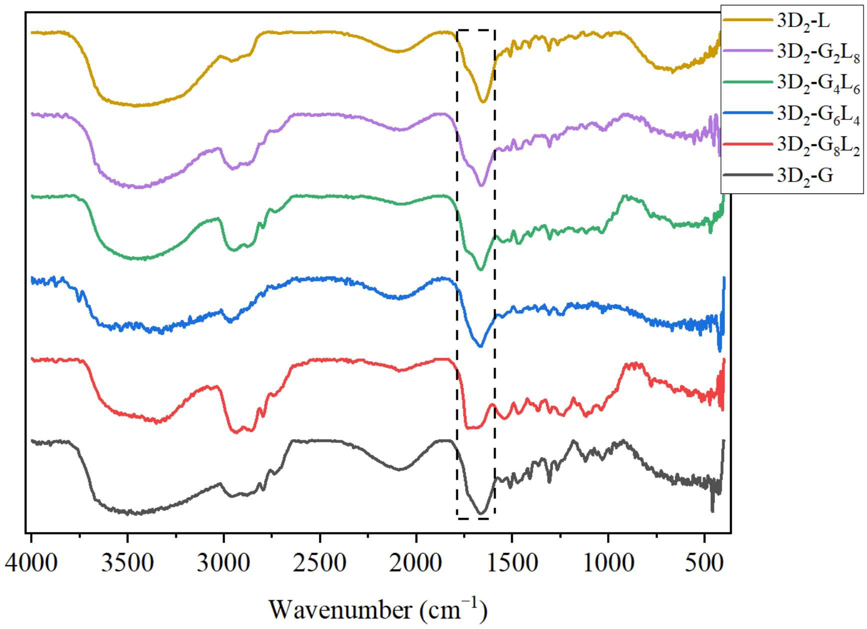

Figure S10. The FTIR spectra of 3D2-G*x*L*y* samples.(where *x*/*y* were 8/2, 6/4, 4/6, and 2/8, respectively. For comparison purposes , also summarizes the results for 3D2-G and 3D2-L.)

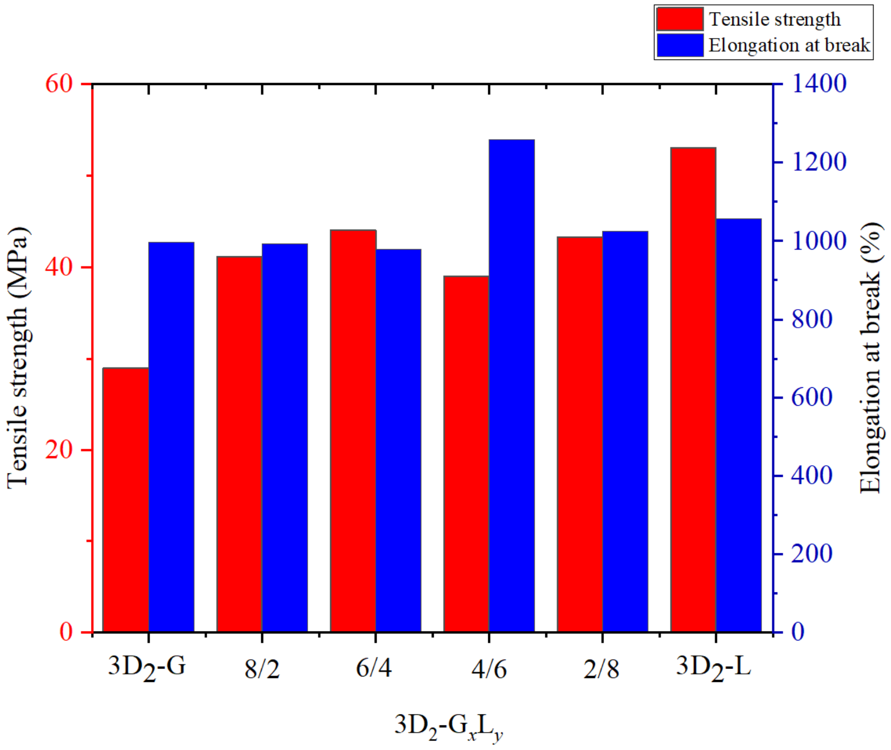

Figure S11. Tensile strength and the elongation at break of $3D_2$-G_xL_y samples.(where *x*/*y* were 8/2, 6/4, 4/6, and 2/8, respectively. For comparison purposes , also summarizes the results for $3D_2$-G and $3D_2$-L.)
